# Discovery and biological evaluation of a diphenethylamine derivative (HS665), a highly potent and selective κ opioid receptor agonist

**DOI:** 10.1186/2050-6511-13-S1-A43

**Published:** 2012-09-17

**Authors:** Mariana Spetea, Ilona P Berzetei-Gurske, Elena Guerrieri, Jayapalreddy Mallareddy, Géza Tóth, Helmut Schmidhammer

**Affiliations:** 1 Department of Pharmaceutical Chemistry, Institute of Pharmacy and Center for Molecular Biosciences, University of Innsbruck, 6020 Innsbruck, Austria; 2 Biosciences Division, SRI International, Menlo Park, CA 94025, USA; 3 Institute of Biochemistry, Biological Research Centre, Hungarian Academy of Sciences, 6701 Szeged, Hungary

## Background

Activation of the κ opioid (KOP) receptor results in antinociceptive actions, while it is not involved in the unwanted effects including respiratory depression, dependence or abuse liability, as in the case of the µ opioid (MOP) receptor. Therefore, KOP agonists appear to possess some advantages over the most widely used MOR analgesics. Besides the analgesic activity, KOP agonists have also shown other beneficial effects such as anti-pruritic, anti-arthritic, anti-inflammatory, and neuroprotective effects. At present, the main classes of available chemically distinct KOP agonists include benzomorphans, morphinans, arylacetamides, diterpenes and peptides. Herein, we present a new molecular scaffold for KOP ligands of the class of diphenethylamines and biological investigations on *in vitro* and *in vivo* opioid activities.

## Methods

Synthesis of the novel KOP ligands was accomplished by multi-step syntheses. Chinese hamster ovary (CHO) cell membranes expressing human opioid receptors were used in radioligand binding and [^35^S]GTPγS functional assays. Antinociceptive activities were assessed in mice using the writhing test.

## Results

Several novel ligands were synthesized and pharmacologically evaluated. Among them, HS665 proved to be the derivative with the highest selectivity for the KOP receptor vs. the other two types, MOP and δ opioid (DOP) receptors (selectivity ratios KOP/MOP >1,000 and KOP/DOP >20,000), and KOP agonist potency. *In vivo*, this derivative produced dose-dependent and significant antinociceptive actions in a mouse model of visceral pain (acetic acid-induced writhing) after subcutaneous administration, being equipotent to the standard KOP agonist U50,488. Antinociceptive effects of HS665 were reversed by the KOP-selective antagonist nor-binaltorphimine demonstrating a KOP receptor-mediated mechanism of the antinociceptive action. HS665 has also recently been prepared in tritium-labeled form ([^3^H]HS665), which can be used as research tool to characterize KOP actions at the cellular and molecular level and to establish the *in vitro* opioid activity profile of new ligands.

## Conclusions

This study shows that through appropriate molecular manipulations, a new class of ligands interacting with KOP has been identified, namely HS665 and derivatives thereof. Such novel KOP ligands, besides the scientific value as pharmacological tools, may also have the potential of emerging as novel therapeutics for human disease states.

